# The X-Rays

**Published:** 1913-02-01

**Authors:** Alfred C. Norman

**Affiliations:** House Surgeon at Durham County Eye Infirmary.


### ELECTRICITY IN MODERN MEDICINE.*

### XXV. The X-Rays (*Concluded*).

By ALFRED C. NORMAN, M.D. Edin., House Surgeon at Durham County Eye Infirmary.

### Advanced X-Ray Work.

Many of the modern refinements of $x$-ray technique belong purely to the domain of the specialist, and their consideration would be out of place in a series of elementary articles such as these. Mention must be made, however, of certain methods (refinements of yesterday) which promise to become ordinary routine in the near future.

### Instantaneous Radiography.

There is now no difficulty in obtaining apparatus that will furnish currents powerful enough to radiograph almost any part of the body in a small fraction of a second, and, when the makers can supply us with tubes at a reasonable price that will stand these enormous currents, there is no doubt that all radiography will be practically instantaneous. At present the method is used chiefly in radiographing parts (such as the heart, or the intestinal tract after a bismuth meal), the movements of which are not under our control, and here it is of the utmost value. Generally speaking, a powerful coil, a special switchboard, and a costly tube are required for instantaneous radiography, but by using celluloid $x$-ray films, and by placing the film between *two* intensifying screens, it is possible to reduce the exposure for most parts of the body to less than one second, even with a twelve-inch coil.

### Orthodiagraphy and Tele-radiography.

The $x$-rays diverge as they proceed from the focus-point on the anticathode, consequently the shadows of objects cast by them are magnified and distorted to a degree depending upon the distances between tube and object and object and plate.

*Orthodiagraphy* consists in making, with the aid of the fluorescent screen, a pencil tracing of the *outline* of an object in its true dimensions. The method was chiefly used to measure the size of the heart, but special and complicated apparatus was required, consequently it was given up by many workers as soon as tele-radiography became possible by virtue of more powerful apparatus.

*Tele-radiography* consists in taking an organ with the tube at such a distance that the rays reaching the plate may, for practical purposes, be regarded as being parallel; consequently the radiogram depicts the organ in very nearly its true dimensions. A distance of 80 inches is usually adopted, and the method is used chiefly to determine the size of the heart. With the anticathode so far from the plate it is necessary to use enormous currents and exceptionally good tubes in order to obtain a sufficiently short exposure.

### Stereoscopic Radiography.

An ordinary radiograph is entirely devoid of perspective, and since, in certain regions of the body this is a great disadvantage, stereoscopic radiography is sometimes employed. Two plates are exposed in succession (care being taken that the position of the patient is identical in each), the tube being moved a short distance in a lateral direction before the second exposure is made. The resulting radiographs are viewed together in a special reflecting stereoscope (or they may be reduced in size and placed in an ordinary stereoscope), when the shadows of parts at varying depths will appear in their relative positions—*i.e.*, in true perspective.

### The Bismuth Meal and the Bismuth Enema.

To supplement ordinary clinical methods in the diagnosis of affections of the alimentary canal the $x$-rays are now used as a matter of routine in most large hospitals, and the very large amount of literature on the subject is a fair index of its growing importance to clinicians. The patient is given a meal or an enema containing some insoluble salt opaque to $x$-rays, and its progress through the intestinal canal is observed on the fluorescent screen. In this method of diagnosis radiography is of very little value, because the whole sequence of events must be taken into consideration, and not one momentary phase. Elaborate apparatus has been designed by certain workers in order that radio-cinematography may be used for this purpose, but the enormous cost will preclude its ever becoming more than a scientific curiosity or—shall we say?—a monument to the genius of its inventors.

Space does not permit of my dealing with alimentary radioscopy at any length in these articles, but much valuable information can be obtained from the writings of such pioneers as Hertz, Kaestle, Rieder, Rosenthal, Haenisch, Cole, Holzknecht, and Schonberg, many of whose papers are to be found in the last three volumes of the "Archives of the Roentgen Ray."

The writer is chiefly indebted to the writings of Dr. Hertz, of Guy's Hospital, for the following practical points.

The nature of the insoluble salt used for the meal is of considerable importance. Bismuth subnitrate is poisonous in large doses and must not be used. Bismuth carbonate, which has held the field for some considerable time and is quite safe even in doses of 8 oz., is becoming unpopular because it neutralises the acidity of the gastric juice, and so diminishes peristalsis. Bismuth oxychloride is free from the above defects, and is now very largely used, but on account of its price will probably be ousted in the near future by barium sulphate. The quantity of the salt used should not be greater than is absolutely necessary, because its weight tends to distort the shape of the stomach and intestines. The meal should be as palatable as possible, otherwise the patient may be so nauseated as to inhibit the

* Previous articles appeared on Nov. 11, 25, Dec. 9, 30, 1911, Jan. 13, 27, Feb. 17, March 9, 30, April 20, May 4, 25, June 8, July 6, Aug. 3, 17, 31, Sept. 28, Oct. 12, 26, Nov. 9, 30, Dec. 14, 1912, and Jan. 11.

normal gastric functions. Dr. Hertz recommends a meal of 2 oz. of bismuth oxychloride mixed with half a pint of well made and sweetened porridge or bread and milk. Normally, such a meal would be evacuated from the stomach in about four hours, but it is very important that nothing further to eat be given until the examination is finished, for a second meal would mix with the bismuth and considerably delay its passage from the stomach. The patient must be prepared with aperients and enemata before a bismuth meal exactly as for an abdominal operation, because an accumulation of fæces would retard the passage of the meal, and so give rise to fallacious inferences.

In investigating the large intestine Dr. Haenisch, of Hamburg, gives an enema consisting of bismuth carbonate, bolus alba, and water. He watches on the fluorescent screen the course of the bismuth from the moment it enters the rectum until it reaches the cæcum, and by noting its behaviour in various abnormal conditions Dr. Haenisch has elaborated a system of differential diagnosis which promises to be of the utmost value in the surgery of the large intestine.

Professor G. von Gourevitsch uses a decoction made from potato flour as a vehicle for his bismuth meals and enemata, and he claims that the results are much more satisfactory than with the old form of meal. Professor von Gourevitsch gives the following formula for his bismuth enema:—Mix two heaped teaspoonfuls of potato flour with 17 oz. of cold water; pour it into 34 oz. of boiling water and stir until it is homogeneous; boil for two minutes. Just before use the bismuth should be added in the following way. Five to ten teaspoonfuls of bismuth is shaken up with 17 oz. of water, and the resulting suspension is carefully stirred into the potato decoction. The temperature of the enema should be 40° C.

### The Dangers of X-Rays.

To read the early *x*-ray literature in the light of our present knowledge is like watching a grim tragedy—the tragedy of pioneers rushing, as it were, blindly to their fate. The enthusiasm of the early workers, their suicidal technique, the first dawn of suspicion gradually growing into certainty, and, finally, in certain cases, the realisation of their inevitable doom, are unfolded before our eyes in a series of papers which, happily, have no parallel in the whole history of medicine.

The pathological results of exposure to *x*-rays may be classified under three headings—namely, acute dermatitis, or *x*-ray "burn"; chronic dermatitis; sterility.

In the early days, when weak induction coils and soft tubes were used, it was no uncommon occurrence for a radiographic exposure to last thirty minutes, or even longer, consequently *x*-ray burns were sometimes inflicted on the patient. Nowadays such an occurrence would amount to criminal negligence.

Chronic dermatitis and sterility are likely to be the lot of an operator who carelessly exposes himself to frequent small doses of the *x*-rays; the effect is cumulative, and the onset of the mischief is so insidious that much harm may be done before the operator realises what is happening. By taking the following precautions, however, every *x*-ray worker can absolutely safeguard himself against any possibility of danger.

The *x*-ray tube must be enclosed in an *x*-ray-proof box fitted with a diaphragm. The operator must never stand in front of the anticathode unless protected by an opaque screen. When a great deal of time is to be spent in the *x*-ray room a lead-lined cabin or else a large lead-glass screen must be fitted up in order to shut off the operator from the part of the room in which the tube is working.

Fluoroscopic examinations are the chief source of danger to the operator, and should not be made except when absolutely necessary. Generally speaking, a radiograph will afford more information than the fluoroscopic image; but when investigating the thorax and in the examination of the abdomen after a bismuth meal, it is necessary to use the fluoroscope, and then special precautions must be taken. The fluoroscope should be covered on the side next the operator by a sheet of lead-glass, and the handles should be guarded by large metal shields. The operator should wear an *x*-ray-proof apron, and (if he does not already wear glasses) a pair of lead-glass spectacles. Above all, it should be made an absolute rule never to test the hardness of the tube by means of the operator's hand—a radiometer will do this far more efficiently.

To safeguard the patient the very soft rays should be cut off by means of a filter (a sheet of aluminium 1 mm. thick, or a piece of sole leather $\frac{1}{4}$ inch thick) placed behind the diaphragm. Prolonged screen examinations must be guarded against. In giving therapeutic doses healthy skin must be protected by lead-foil or by a special *x*-ray-proof paste.

*(To be continued.)*

